# Mitochondrial Cyt b Reveals Low Diversity and Basin-Scale Population Structure in Black Carp (*Mylopharyngodon piceus*) from the Yangtze, Pearl and Red River Basins

**DOI:** 10.3390/ani16050768

**Published:** 2026-03-01

**Authors:** Yan-Qiao Li, Xing-Pu Huang, Dan Li, Tong Wu, Xiao-Yan Fu, Yu-Ning Zhang, Qi Huang, Gui-Feng Wei, Ling-Lin Wan, Qun Zhang

**Affiliations:** 1 Department of Ecology and Institute of Hydrobiology, Jinan University, Guangzhou 510632, China; yanqiao.liii@outlook.com (Y.-Q.L.); 18933794228@163.com (X.-P.H.); yisumonky@foxmail.com (T.W.); txyfu66@163.com (X.-Y.F.); q190307@163.com (Y.-N.Z.); 15521302416@163.com (Q.H.); tweigf@jnu.edu.cn (G.-F.W.); linglinwan@jnu.edu.cn (L.-L.W.); 2 Institute of Water Science, Guangzhou 510220, China; danylee@126.com

**Keywords:** *Mylopharyngodon* *piceus*, population genetics, population decline, cytochrome b, genetic differentiation, genetic diversity, conservation

## Abstract

The black carp (*Mylopharyngodon piceus*) is an ecologically and economically important freshwater fish native to China and neighbouring regions, but its wild stocks have declined sharply in recent decades. We analysed mitochondrial cytochrome b (Cyt b) sequences from 100 individuals collected in 2008–2009 from four Yangtze River, two Pearl River and one Red River populations to assess genetic diversity and structure as a pre-ban baseline for maternal lineages. Sixteen polymorphic sites defined 17 haplotypes, with a single dominant haplotype (Hap2) shared across all populations. Haplotype diversity was high but nucleotide diversity low, and neutrality tests together with mismatch-distribution analyses were consistent with a recent Late Pleistocene demographic expansion. Pairwise FST values ranged from negligible differentiation among middle–lower Yangtze populations to pronounced differentiation between the upstream Yangtze population (SS) and middle–lower populations and between the Yangtze and the combined Pearl–Red basins, whereas Pearl and Red River populations showed no significant divergence and high mitochondrial homogeneity, consistent with substantial historical connectivity. Overall, the Cyt b data indicate low mitochondrial diversity and shallow but significant inter-basin structuring, providing preliminary mtDNA-based evidence that Yangtze and Pearl–Red populations represent candidate conservation and management units, and highlighting the need for nuclear genomic markers and contemporary sampling to refine drainage-scale units and evaluate recent management effects.

## Simple Summary

Black carp is a large freshwater fish native to East Asia that feeds mainly on snails and clams, helping to control pests and maintain river ecosystems. However, wild stocks have declined because of overfishing, river regulation, pollution and poorly regulated stocking practices. Here, we analysed a mitochondrial DNA marker (cytochrome b, Cyt b) in fish sampled at seven sites in the Yangtze, Pearl and Red River basins during 2008–2009. These pre-ban samples provide a snapshot of maternal lineages before the ten-year fishing ban in key reaches of the Yangtze River and before recent adjustments to stocking practices. We found generally low mitochondrial genetic diversity, especially in the Pearl and Red River groups, which may limit the capacity of local populations to cope with environmental change and disease. Yangtze populations werve more diverse but already differed between the middle–lower Yangtze and an upstream site and showed broad separation from the combined Pearl–Red River group. These patterns suggest that uncritical movements of broodstock or juveniles among basins could erode remaining genetic differences. Protecting key habitats, using genetically diverse, locally adapted broodstock and avoiding unnecessary mixing among basins will help conserve black carp and support more sustainable river fisheries.

## 1. Introduction

The black carp (*Mylopharyngodon piceus*) is a key component of Chinese freshwater fisheries owing to its delicate flesh and high nutritional value [1]. As a large, molluscivorous cyprinid of the family Xenocyprididae in the order Cypriniformes, it occupies a relatively high trophic level [2,3,4], regulates benthic mollusc populations and helps maintain food-web structure [5]. Its highly specialised diet on snails, clams and mussels requires specific habitats and constrains its natural distribution. The black carp is native to the Yangtze, Pearl and Yellow Rivers in China and to the Red River along the China–Vietnam border and occurs only sporadically in the Heilongjiang (Amur) River. Despite widespread aquaculture introductions, its abundance in natural waters is lower than that of the other three major Chinese carp species, and its naturally small wild population sizes make recovery more difficult than in filter-feeding species such as *Ctenopharyngodon idella*, *Hypophthalmichthys molitrix* and *Aristichthys nobilis*. This not only threatens the persistence of black carp but may, through trophic cascades, affect freshwater ecosystem structure and functioning.

Overfishing, water pollution, hydraulic engineering, habitat fragmentation and destructive practices such as electrofishing and dynamite fishing have jointly accelerated the decline of wild stocks [6,7,8]. Monitoring has shown that, compared with the 1990s, reproductive stocks of the four major Chinese carp species in the middle–lower Yangtze River have declined markedly, with the mean annual abundance of drifting eggs and larvae decreasing from 2.043 to 1.688 billion individuals [9]. Large-scale hatchery-based stock enhancement has been widely implemented, but narrow broodstock sources, inbreeding and poorly regulated releases have eroded the genetic integrity of wild populations and led to genetic deterioration of hatchery strains, thereby reducing genetic diversity and adaptive potential and threatening both sustainable fishery development and ecosystem health [10]. Among the four major Chinese carp species, black carp has the lowest natural stocks and the weakest research foundation; studies on its population biology and genetic diversity are fewer than for the other three species [11,12,13]. This lag in research and conservation is disproportionate to its importance for fisheries and ecosystem functioning, underscoring the need for a systematic assessment of its current genetic background and for science-based conservation and management strategies.

Although relatively few studies have examined black carp population genetics, several works provide important context [14,15,16]. Li et al. [17] compared mitochondrial D-loop, Cyt b and nuclear markers among all four major Chinese carp species in the middle Yangtze River and found that black carp showed intermediate genetic diversity. Tang et al. [18] analysed complete mitochondrial genomes of wild and farmed black carp from several basins and reported overall low mitochondrial nucleotide diversity (π ≈ 0.0010–0.0024), moderate to high differentiation and restricted gene flow among populations, with neutrality tests and mismatch distributions indicating a historical bottleneck followed by expansion. Bao et al. [19] described the complete mitochondrial genome of a grey black carp individual from the Pearl River, providing a reference mitogenome but limited population-level information. In contrast, microsatellite data for *C. idella* revealed very low differentiation and high connectivity among the Yangtze, Pearl and Heilongjiang Rivers [20], whereas black carp appears to exhibit stronger genetic structuring between Yangtze and Pearl River populations, although its overall phylogeographic pattern remains unclear [21].

Marker heterogeneity and differences in evolutionary rates among gene fragments can lead to inconsistent inferences [22,23], and most existing studies have been constrained by limited population coverage and heterogeneous markers, resulting in an incomplete picture of black carp genetic resources [24,25]. Here, we use the mitochondrial cytochrome b (Cyt b) gene, a moderately long protein-coding marker with a relatively conserved substitution rate, robust amplification and good comparability among studies [26,27,28]. Cyt b retains sufficient variation to resolve divergence among closely related populations and is widely used in fish species identification and population genetic analyses [29,30,31,32]. Because Cyt b is maternally inherited, our study primarily reflects the spatial structure of mitochondrial lineages and should be viewed as a preliminary, drainage-scale depiction of the maternal genetic pattern of black carp, to be refined by future work incorporating nuclear and genomic markers.

In this study, we obtained complete Cyt b sequences from seven representative populations spanning two major Chinese rivers (Yangtze and Pearl) and the Red River system along the China–Vietnam border. All samples were collected in 2008–2009, prior to implementation of the ten-year fishing ban in key reaches of the Yangtze River and during a period of intensive stock enhancement; our data therefore approximate a “pre-ban” mitochondrial baseline rather than contemporary post-2020 structure. By combining a larger sample size with broader spatial coverage than most previous studies, we aimed to reduce sampling bias and avoid overly restricted conclusions. Specifically, we sought to characterise within-population genetic diversity, among-population and among-basin differentiation, and demographic history of black carp, and to evaluate the relative roles of natural geography and human activities in shaping current patterns. These results provide a scientific basis for the conservation and rational utilisation of black carp genetic resources [33,34].

## 2. Materials and Methods

### 2.1. Sample Collection

**Figure 1:**
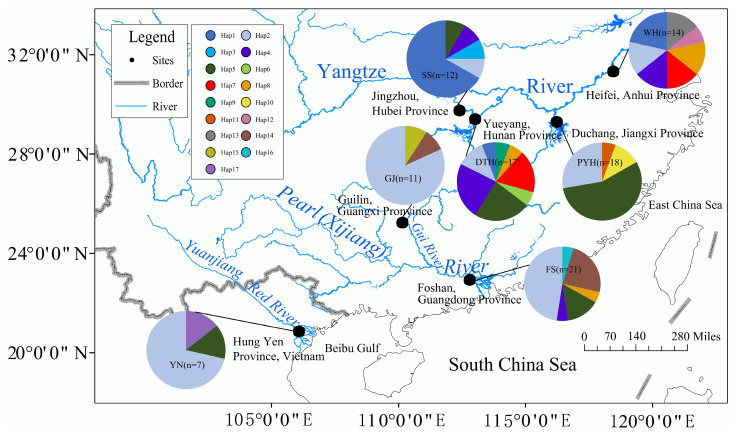
Map of the study area. Sampling localities and mitochondrial Cyt b haplotype composition of black carp in the Yangtze, Pearl (Xijiang) and Red River basins. Pie charts at each site show the proportional frequencies of haplotypes, with colours denoting different haplotypes as indicated in the figure legend. Site codes (SS, DTH, PYH, WH, GJ, FS, YN) correspond to those in Table 1.

Black carp were sampled in 2008–2009 from catches obtained by local fishers using gill nets, cast nets and fyke nets at six sites in the Yangtze and Pearl River basins, China (Figure 1; Table 1). The material comprised 12 individuals from Shishou (SS) and 17 from Dongtinghu (DTH) in the middle Yangtze, 18 from Poyanghu (PYH) in the middle–lower Yangtze, 14 from Wuhu (WH) in the lower Yangtze, 11 from the Guijiang River (GJ; a tributary of the Pearl River) and 21 from Foshan (FS) in the Pearl River mainstem. All individuals were identified to species using standard morphological keys and preserved in 95% ethanol. Because black carp is not listed as an endangered or protected species and sampling was conducted in public waters under local fishery regulations, no additional collection permits were required. All specimens were collected in compliance with Chinese law, and procedures were approved by the Animal Ethics Committee of the Department of Ecology, Jinan University (JNU2024012), following institutional and national guidelines for the care and use of animals.

In addition, seven Cyt b sequences from black carp in the Red River basin, Vietnam (population YN), were retrieved from GenBank and included in the analyses. For this population, only the metadata accompanying the original submissions were available, but the sequences had passed standard GenBank quality control. Given the small sample size and external origin of this data set (*n* = 7), inferences involving YN were treated as tentative, and key analyses were repeated with YN excluded to assess the robustness of the main results.

### 2.2. Genomic DNA Extraction

Genomic DNA was extracted from the muscle tissue of each specimen. Briefly, an appropriate amount of muscle was incubated in digestion buffer containing proteinase K at 37 °C until complete lysis. DNA was purified by phenol–chloroform extraction. The lysate was mixed with 200 μL Tris-saturated phenol (lower phase) and 200 μL chloroform, centrifuged (13,000 rpm, 10 min), and the upper phase (~400 μL) was transferred to a new tube. After a second chloroform cleanup (+600 μL chloroform; 13,000 rpm, 10 min), ~200–300 μL of the upper phase was collected, and DNA was precipitated with 600 μL ice-cold 95% ethanol at −20 °C for 2 h. The DNA pellet was recovered by centrifugation (13,000 rpm, 10 min), washed twice with 600 μL 70% ethanol (13,000 rpm, 5 min each), air-dried (room temperature overnight or 27–37 °C for 1–2 h), resuspended in 40 μL TE, incubated at 4 °C for 2–3 h to allow complete dissolution and stored at −20 °C [35].

### 2.3. Amplification and Sequencing

The target Cyt b fragment was amplified by PCR using a newly designed, species-specific primer pair for black carp, Cyt bL (5′-ACC GAG ACC AAT GAC TTG AAR AAC CAC CGT TG-3′) and Cyt bR (5′-CTT TGG GAG TTA GGG GTG GGA G-3′). Each 20 μL reaction contained 10.0 μL of 2× premix (Taq DNA polymerase, buffer and dNTPs), 7.0 μL ddH_2_O, 1.0 μL of each primer (10 μM) and 1.0 μL template DNA. The thermal profile consisted of 94 °C for 5 min; 34 cycles of 95 °C for 30 s, 55 °C for 30 s and 72 °C for 90 s, and a final extension at 72 °C for 7 min, followed by a 4 °C hold.

PCR products were checked on 1% agarose gels, and amplicons of the expected size were sequenced in the forward direction on an automated capillary sequencer (Guangzhou Tianyihuiyuan Gene Technology Co., Ltd., Guangzhou, China). Chromatograms generally showed single, well-resolved peaks, allowing reliable consensus sequences to be obtained from forward reads; sequences with ambiguous or low-quality calls were re-sequenced or discarded. Resulting Cyt b sequences were manually edited and assembled in BioEdit 7.0.9.0, and their identity and integrity were verified by NCBI BLAST ver 2.17.0 searches (National Center for Biotechnology Information, Bethesda, MD, USA) against the NCBI database. Only high-quality, full-length Cyt b sequences were retained for subsequent analyses.

### 2.4. Data Analysis

The sequenced fragments were manually edited and aligned in BioEdit 7.0.9.0 [36]. MEGA 7.0 [37] was used to calculate nucleotide composition, base frequencies, numbers of variable sites and the transition/transversion profile. DnaSP 6.12.03 [38] was used to estimate the number of haplotypes, haplotype diversity (Hd) and nucleotide diversity (π) [39], and to perform mismatch-distribution analyses. Geographic haplotype patterns were visualised as site-specific haplotype-frequency pie charts (Figure 1), and a haplotype network was constructed in PopArt 1.7 [40]. Tajima’s D [41], Fu’s Fs [42], the sum of squared deviations (SSD), Harpending’s raggedness index (Rg) and the expansion parameter τ [43,44] were calculated to test for departures from neutrality and for fit to a sudden demographic expansion model.

Pairwise FST [45] and analysis of molecular variance (AMOVA) [46] were computed in Arlequin 3.5.2.2 [47] with 1000 permutations. Spatial analysis of molecular variance (SAMOVA 2.0) was run for K = 2–6; the smallest K with a significant among-group component (FCT) and geographically coherent groupings (K = 2) was then adopted as an additional AMOVA grouping scheme. The effective number of migrants per generation (Nm) was estimated from FST under the island model [48] as Nm = (1 − FST)/(4FST) for positive FST values, whereas negative FST values, interpreted as indicating no detectable differentiation due to sampling variance, were not converted. Because the Red River population (YN) is represented by a small external GenBank data set (*n* = 7), inferences involving this population were treated as tentative, and global FST and AMOVA were also recalculated without YN, yielding similar patterns. Pairwise FST and Nm matrices were visualised using metric multidimensional scaling (MDS) and heatmaps, truncating negative FST values to zero and keeping a consistent population order, with the MDS plots and heatmaps generated in Python 3.11.2 using NumPy 1.24.0 and Matplotlib 3.7.5 (Python Software Foundation, Beaverton, OR, USA).

The time since population expansion (T) was estimated as T = (τ/2μk) × generation time, where τ is the expansion parameter, μ is the substitution rate per site per year, and k is sequence length. A commonly used Cyt b substitution rate of 1–2% per million years for cyprinid fishes was adopted to obtain an order-of-magnitude estimate of expansion time [49,50].

## 3. Results

### 3.1. Cyt b Sequence Variation and Genetic Diversity in Black Carp

After sequencing and editing, 100 complete Cyt b gene sequences of black carp, each 1140 bp in length, were obtained. Across these sequences, the proportions of T, C, A and G were 26.9%, 28.4%, 30.9% and 13.9%, respectively. The A+T content (57.8%) was slightly higher than the C+G content (42.3%), indicating an A+T-rich composition consistent with most fish mitochondrial genomes. The transition/transversion ratio (Ts/Tv) was 2.9, showing that substitutions were dominated by transitions. In total, 16 variable sites were detected across the 1140 bp alignment, of which 14 were parsimony-informative, defining 17 haplotypes.

Genetic diversity indices for the seven geographic populations and the three major river systems (the Yangtze, Pearl and Red Rivers) are summarised in Table 2. Haplotype diversity in the Yangtze River system was slightly higher than in the Pearl and Red River systems. Among the seven populations, haplotype diversity ranged from 0.345 to 0.912, with the WH population in the lower Yangtze exhibiting the highest value (Hd = 0.912), the GJ population in the Pearl River showing the lowest value (Hd = 0.345), and the remaining populations showing moderate to high diversity (Hd > 0.6). In contrast, nucleotide diversity was low in all populations (π = 0.0005–0.0025), indicating relatively limited intraspecific mitochondrial variation at this locus. Overall, black carp showed the characteristic mitochondrial pattern of high haplotype diversity (Hd) combined with low nucleotide diversity (π).

### 3.2. Haplotype Network of Black Carp

**Figure 2:**
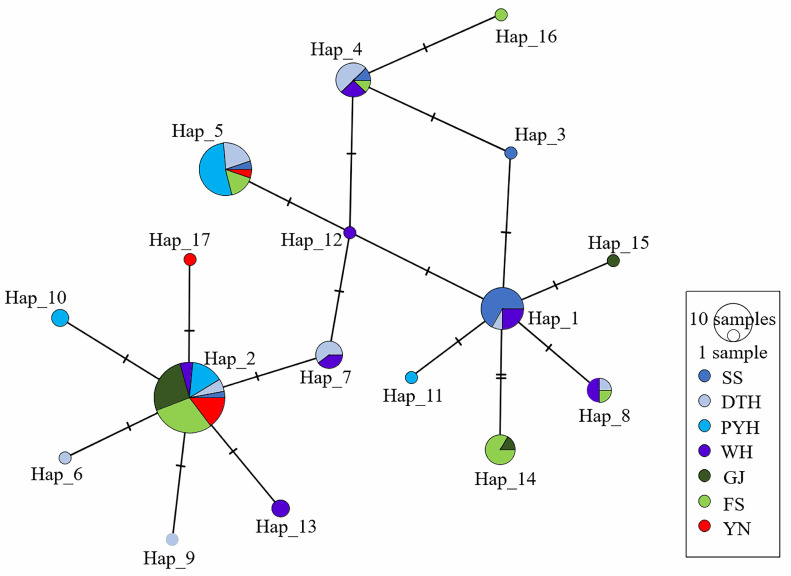
Haplotype network based on mitochondrial Cyt b sequences of black carp. Each circle represents a distinct haplotype, with circle size proportional to the number of individuals. Colours indicate sampling populations (SS, DTH, PYH, WH, GJ, FS and YN), as shown in the legend. Labels (Hap_1–Hap_17) next to the circles denote haplotype IDs. Small black dots represent inferred but unsampled intermediate haplotypes. Short hatch marks on branches indicate single mutational steps.

The Cyt b haplotype network based on the 17 haplotypes (Figure 2) was shallow and approximately star-like, with a maximum of five mutational steps among haplotypes and only a few short branches. Haplotypes from different basins were extensively intermingled, with only weak geographic clustering; several Yangtze haplotypes (e.g., Hap2, Hap5) grouped together with haplotypes from the Pearl and Red River basins. The most frequent haplotype, Hap2, occurred in all populations. Consistent with the network, the geographic distribution of haplotype frequencies (Figure 1) showed that Hap2 and a few other common haplotypes were shared across all three basins but at clearly different frequencies, whereas many low-frequency haplotypes were restricted to single basins or even single populations. Taken together, these patterns indicate low mitochondrial sequence divergence and no deeply separated lineages, consistent with a shallow mitochondrial genealogy shaped by recent demographic history, with basin-specific differences in haplotype composition and frequency superimposed.

### 3.3. Population Genetic Differentiation of Black Carp

**Figure 3:**
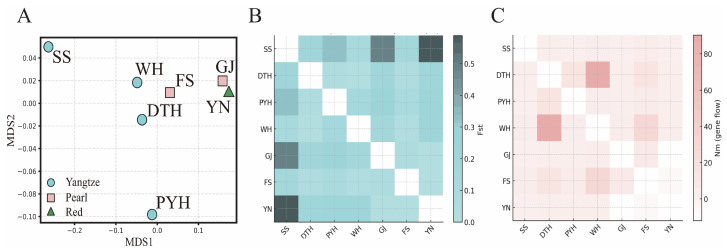
Multidimensional scaling and heatmaps of pairwise mitochondrial parameters in seven black carp populations. (**A**) Metric multidimensional scaling (MDS) based on pairwise Cyt b FST; populations are symbol- and colour-coded by river basin (Yangtze, Pearl, Red). (**B**) Heatmap of pairwise genetic differentiation (FST; values below the diagonal in Table 3; negative estimates set to zero). (**C**) Heatmap of approximate gene flow (Nm; values above the diagonal in Table 3). In both heatmaps, populations are ordered identically, darker colours indicate higher FST or Nm, lighter colours lower values, and diagonal cells are blank.

Genetic differentiation coefficients, FST-based estimates of gene flow and AMOVA results (Table 3 and Table 4), together with the MDS ordination (Figure 3A), indicate that black carp exhibits moderate drainage-associated structuring of mitochondrial haplotype frequencies, while most mitochondrial variation is retained within populations. Pairwise FST values among the seven populations ranged from −0.071 to 0.589 (Table 3), spanning a continuum from effectively no differentiation (FST ≈ 0) to high differentiation (FST ≥ 0.25) in the qualitative sense commonly used for FST.Genetic differentiation among the three middle–lower Yangtze populations was low (FST < 0.05), whereas the upstream SS population was clearly more differentiated from the two downstream Yangtze populations (FST > 0.15). The GJ population from the Pearl River showed FST > 0.15 with all four Yangtze populations, all significant or highly significant, and the FS population also differed significantly from some Yangtze populations. Likewise, FST between the Red River population YN and each Yangtze population exceeded 0.20 and was highly significant, indicating marked mitochondrial differentiation between YN and the Yangtze populations at this locus. In the MDS plot, the three middle–lower Yangtze populations (DTH, PYH and WH) cluster closely, SS occupies a more distant position, and the two Pearl River populations (GJ and FS) lie near YN, forming a south-western group. This configuration summarises the pairwise FST patterns and highlights a primary axis of differentiation between the Yangtze basin and the combined Pearl–Red basins, together with additional differentiation of the upstream Yangtze population SS.

The geographic distribution of haplotype frequencies (Figure 1) is consistent with these patterns. Yangtze populations harbour a relatively rich set of haplotypes, including several low-frequency variants, whereas Pearl and Red River populations share most of their haplotypes with the Yangtze but show reduced haplotype richness and marked shifts in the frequencies of common haplotypes (e.g., Hap2). Thus, the significant FST values mainly reflect differences in haplotype frequencies and the presence or absence of rare haplotypes among basins rather than deeply divergent, basin-specific mitochondrial lineages. This reconciles the shallow, weakly structured haplotype network (Figure 2) with the moderate basin-scale differentiation detected by FST, MDS and AMOVA.

In contrast, FST values between the two Pearl River populations and the Vietnamese Red River population were close to zero, indicating high mitochondrial genetic homogeneity; at Cyt b, these three populations show no detectable differentiation and can be regarded as forming a single, weakly structured cluster. Pairwise estimates of gene flow (Nm) among the seven populations (Figure 3) exceeded 1 for most population pairs, suggesting non-trivial historical genetic connectivity as approximated from FST-based Nm, while recognising the well-known limitations of inferring absolute migration rates from this statistic. In the MDS and Nm heatmaps, a low-differentiation cluster centred on the middle–lower Yangtze appears relatively connected to the Pearl River, whereas edge populations such as SS and YN are more strongly differentiated.

AMOVA further supported this interpretation. When all seven populations were analysed together, 85.14% of the total variation occurred within populations and 14.86% among populations (overall FST = 0.149, *p* < 0.001). Grouping populations by the three river systems yielded only 5.82% of the variance among basins (FCT = 0.058), and a two-group partition (Yangtze versus the combined Pearl–Red group) increased the among-group component only slightly to 7.53% (FCT = 0.075; global FST = 0.179, *p* < 0.001), consistent with the MDS contrast between the Yangtze and the southern edge basins. Excluding YN produced similar results (global FST = 0.133; among-population variance = 13.3%), and the SAMOVA grouping that combined GJ and YN yielded the highest, but still modest, among-group component (14.60% of the variance; FCT = 0.088, *p* < 0.05). Overall, these analyses emphasise that most mitochondrial variation resides within populations rather than among drainage-based groups, with shallow but statistically significant basin-scale structuring superimposed on this within-population diversity.

### 3.4. Demographic History of Black Carp

Neutrality tests and mismatch-distribution analyses were used to infer the demographic history of black carp. For the pooled sample, Tajima’s D was negative (−0.655) but did not differ significantly from zero, whereas Fu’s Fs was significantly negative (−5.218), consistent with an excess of low-frequency haplotypes and a recent increase in effective population size. The mismatch-distribution statistics were small and non-significant (SSD = 0.021, Rg = 0.076), indicating that the observed distribution does not deviate from the sudden demographic expansion model. Taken together with the overall pattern of high haplotype diversity and low nucleotide diversity, these results support a scenario in which black carp underwent a relatively recent expansion following a genetic bottleneck or founder event, rather than maintaining a long-term stable neutral equilibrium.

**Figure 4:**
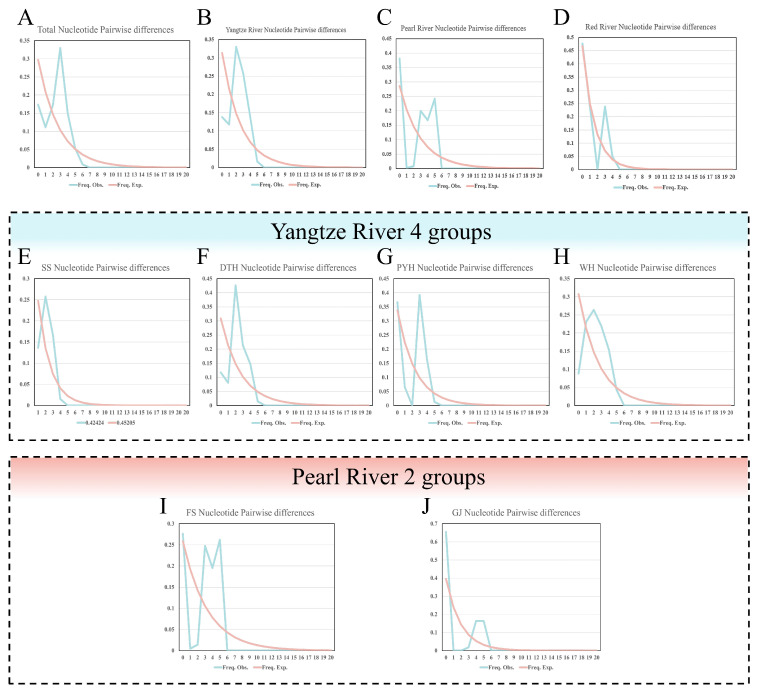
Nucleotide mismatch distribution curves ((**A**): Total, (**B**): Yangtze River, (**C**): Pearl River, (**D**): Red River, (**E**): SS, (**F**): DTH, (**G**): PYH, (**H**): WH, (**I**): FS, (**J**): GJ).

The overall nucleotide mismatch distribution was unimodal (Figure 4), with an estimated expansion parameter τ ≈ 2.971. Assuming a commonly used Cyt b substitution rate of 1–2% per million years for cyprinid fishes, this τ corresponds to an approximate expansion time on the order of 7.0 × 10^4^–1.5 × 10^5^ years before present. Because a species-specific calibration is lacking and the mitochondrial molecular clock may vary by at least a factor of two, this estimate should be interpreted as an order-of-magnitude approximation rather than a precise date; here we simply note that the inferred expansion falls within the Late Pleistocene, consistent with many fish mtDNA studies that place demographic expansions in this interval.

At the drainage scale, the Yangtze and Red River groups showed broadly unimodal mismatch curves, consistent with a dominant expansion episode, whereas the Pearl River group showed a more complex, multimodal pattern, compatible with a demographic history involving multiple bottlenecks and/or expansions. Within basins, mismatch distributions also varied among populations (e.g., broad, low unimodal curves in the low-diversity SS population, sharper unimodal peaks in DTH and irregular multimodal curves in GJ and FS), reinforcing the view that demographic histories have differed among local populations. However, these inferences remain tentative given the single-locus mitochondrial dataset and modest sample sizes and should be re-evaluated using nuclear genomic data and additional temporal and spatial sampling.

## 4. Discussion

### 4.1. Population Genetic Structure and Phylogeographic Pattern of Black Carp

The Cyt b haplotype network reveals a shallow mitochondrial genealogy dominated by a widely shared haplotype (Hap2) and numerous low-frequency haplotypes restricted to single basins or populations. Together with the geographic haplotype-frequency map, this indicates that most populations share common maternal lineages but differ in the frequencies of common haplotypes and in the presence or absence of rare ones. This configuration is consistent with a relatively recent demographic expansion of black carp, likely overlain by human-mediated hatchery-based stock enhancement, as reported for other stocked cyprinids with star-like mtDNA networks and basin-specific shifts in haplotype frequencies [51,52,53]. Large-scale releases in the Yangtze, Pearl and other rivers, often without strict use of local broodstock, are expected to promote admixture among basins and generate shared haplotypes across drainages. The bimodal mismatch distribution and relatively high haplotype diversity in PYH may reflect contributions from broodstock of mixed geographic origin, although this remains speculative.

Despite overall low mitochondrial variation, our results show moderate but significant population structure partly aligned with drainage boundaries. Within the Yangtze, middle–lower populations are closely connected and can function as a single reproductive unit when the mainstem and floodplain lakes are well linked, consistent with life-history traits such as adhesive demersal eggs and spawning migrations within historically continuous river–lake systems [54,55,56]. By contrast, SS in the middle–upper Yangtze is clearly differentiated from DTH, PYH and WH, suggesting reduced connectivity and stronger local drift upstream. Restricted hydrological exchange between middle–upper and middle–lower reaches, together with topographic barriers between the Yangtze and Pearl–Red systems, likely limits movement among regions. The FST heatmap and MDS ordination summarise these relationships: DTH, PYH and WH form a tight middle–lower Yangtze cluster; SS occupies a more peripheral position; and the two Pearl River populations (GJ, FS) cluster with the Red River population (YN), forming a south-western group distinct from most Yangtze samples. At Cyt b, Pearl and Red River populations show high mitochondrial similarity and no detectable differentiation, consistent with substantial historical connectivity, although the small YN sample precludes firm inference about true panmixia [57,58,59].

No deep, basin-specific mitochondrial lineages were detected, yet population differentiation remains moderate and statistically robust, with clear differences in haplotype frequencies among river systems and regions. This pattern likely reflects the joint influence of species-level demographic history and more recent human impacts. Black carp may historically have formed a single lineage originating, together with *Ctenopharyngodon idella* and *Hypophthalmichthys molitrix*, in south-central China, such that deep mitochondrial divergence among basins has not accumulated, while stocking and translocations have further weakened or redistributed some geographic differences [60,61]. Under this view, the apparent discrepancy between the shallow, weakly structured haplotype network and the significant FST estimates is resolved: mitochondrial lineages are recently derived and widely shared, but their relative frequencies and the occurrence of rare haplotypes differ among basins and regions. As illustrated by the haplotype-frequency map, such frequency shifts can generate moderate FST and AMOVA signals even when genealogical divergence is low, a pattern also reported in other riverine cyprinids with recent expansion and stocking histories [51,53,62,63]. Consistent with this interpretation, variance-partitioning analyses indicate that mitochondrial lineages are broadly shared across space and that drainage-associated structure reflects relatively shallow historical and contemporary processes rather than long-standing isolation of deeply divergent lineages. If wild black carp continue to experience reduced connectivity, this tendency towards increased differentiation is likely to intensify, underscoring the need to maintain or restore natural connectivity where feasible and to manage stocking carefully to avoid further perturbation of regional population structure.

### 4.2. Demographic Dynamics of Black Carp Populations

Across its range, black carp exhibits relatively high haplotype diversity but low nucleotide diversity, unevenly distributed among basins. This high-Hd/low-π pattern, widely interpreted in fish population genetics as indicative of demographic expansion from reduced ancestral populations, has been documented in many freshwater and marine fishes [64,65,66]. In our data, the Yangtze populations are more diverse than those in the Pearl and Red River systems, consistent with the breadth and environmental heterogeneity of the Yangtze mainstem and a larger long-term effective population size. Historical records likewise indicate that numerous middle–lower Yangtze lakes have long served as major spawning and nursery areas where black carp were abundant; fishery monitoring by Gao et al. showed that although early life-stage resources of the four major Chinese carps in the Yangtze declined sharply by the late 20th century, the Yangtze remained a principal distribution area for black carp [9].

By contrast, Pearl and Red River populations generally show reduced mitochondrial diversity, consistent with stronger bottlenecks and smaller long-term effective sizes, potentially linked to stocking, habitat alteration and regional population declines, although these drivers cannot be disentangled from the present data. Within this southern group, the GJ population in the Pearl River shows the lowest diversity, whereas the YN population at the southern range margin, historically connected to the Pearl system, has slightly higher but still reduced diversity [67]. Overall, mitochondrial variation in black carp appears limited, plausibly reflecting a combination of long lifespan, extended generation time, relatively small long-term effective size and recent human-driven declines, as also suggested for other long-lived freshwater fishes with restricted distributions.

The relatively low genetic diversity pattern of black carp, together with the significantly negative Fu’s Fs for the total sample, broadly unimodal mismatch distributions for the combined dataset and for the Yangtze and Red River groups, and the coalescent-based time estimates, collectively point to a demographic expansion within a Late Pleistocene time window. However, the absolute timing of this expansion remains uncertain because it is inferred from a single mitochondrial locus and from a substitution rate extrapolated from other cyprinids rather than a species-specific calibration and should therefore be regarded as an order-of-magnitude approximation.

Superimposed on this shared expansion signal, basin-level patterns indicate heterogeneous demographic histories, particularly in the south. In the Pearl River, more complex or weakly multimodal mismatch curves suggest departures from a simple, single-epoch expansion and are consistent with more complex scenarios involving repeated bottlenecks, secondary expansions or stocking. Comparable cases, in which mismatch-distribution complexity reflects interactions among climate-driven range shifts, local bottlenecks and human impacts, have been reported for other freshwater fishes [68,69]. Given that mismatch distributions are sensitive to sampling design, underlying population structure and stocking history, these basin- and population-specific interpretations should be regarded as tentative working hypotheses that highlight demographic complexity rather than definitive reconstructions of glacial–interglacial dynamics.

### 4.3. Conservation and Management of Black Carp

The main mitochondrial contrast detected here is between Yangtze populations and those from the combined Pearl–Red basins, with additional differentiation of the upstream Yangtze population SS. In line with the concept of genetically informed management units, we regard the Yangtze and Pearl–Red groups as provisional, mtDNA-defined candidate management units, and SS as a particularly distinct upstream unit within the Yangtze. However, robust delineation of conservation and management units should integrate nuclear and genomic markers as well as demographic information, and these hypothesis-based units should be confirmed or revised with more extensive, contemporary sampling before they are used to guide formal regulations or basin-scale harvest policies.

In practical terms, our results support a precautionary stance towards large-scale translocations of broodstock or juveniles across basin boundaries and indicate that within-basin actions such as genetic rescue, broodstock supplementation or stock enhancement should be guided by dedicated genomic assessments rather than Cyt b patterns alone. The middle–lower Yangtze, particularly the WH population, appears to harbour the highest mitochondrial diversity and connectivity and may act as an important reservoir of maternal lineages; protecting this diversity and considering WH as a potential broodstock source should await concordant evidence from nuclear genomic data. By contrast, the low-diversity SS population emerges as especially vulnerable; reducing overfishing and habitat disturbance and, where feasible, restoring hydrological and genetic connectivity with downstream reaches may help slow further loss of diversity. In the Pearl River and in the small edge population YN in the Red River, complex or low-diversity patterns primarily act as warning signals that uncritical stocking and introductions could homogenise or disrupt local genetic composition, underscoring the need for strengthened field sampling, stricter control of non-local or poorly documented hatchery stocks and, in the transboundary Red River, cross-border coordination before any assisted gene flow is implemented.

Given that the present dataset is restricted to a single maternally inherited marker, limited spatial and temporal coverage and a small external Red River sample, the conservation recommendations above should be regarded as precautionary and hypothesis-generating rather than prescriptive. Nevertheless, they are consistent with widely accepted genetic guidelines for stocking and hatchery management, including the use of broad and locally based broodstock collections, avoidance of narrow broodstock bases or reliance on single hatcheries, maintenance of sufficiently large effective broodstock sizes, minimisation of the proportion of hatchery-origin fish in natural spawning runs and implementation of pre-release genetic screening and post-release genetic monitoring to reduce risks of inbreeding, outbreeding depression and erosion of the genetic integrity of wild populations. Applying these principles can help reduce immediate genetic risks while more comprehensive genomic and demographic data are gathered to develop species- and basin-specific management strategies for black carp.

## 5. Conclusions

As a species of high ecological and economic importance, black carp remains of clear conservation concern. By analysing mitochondrial Cyt b sequences from specimens collected in 2008–2009, i.e., before the implementation of recent conservation policies such as the ten-year fishing ban in key reaches of the Yangtze River and before several adjustments to stocking programmes, this study provides an mtDNA-based pre-ban baseline for maternal lineages in the Yangtze, Pearl and Red River basins, rather than a complete description of contemporary genome-wide genetic patterns. Against this historical backdrop, the mtDNA data reveal generally low mitochondrial genetic diversity and moderate, basin-associated population differentiation. They further provide preliminary evidence that Yangtze populations and those from the combined Pearl–Red River basins may form two candidate management and conservation units that merit differentiated protection strategies, subject to validation and potential revision by nuclear genomic markers and more extensive, contemporary sampling.

More broadly, the current mitochondrial evidence, together with previous genetic studies on the four major Chinese carp species, suggests that black carp may possess standing genetic diversity that is comparable to, or somewhat lower than, that of the other three major Chinese carps, although this inference requires formal comparative genomic assessment. Even moderate basin-scale structuring could be further eroded by uncritical translocations and hatchery-based stock enhancement. Rather than prescribing specific management interventions, the present results are best viewed as an initial drainage-scale genetic baseline and a set of testable hypotheses. These hypotheses should be re-evaluated with genome-wide markers and up-to-date field sampling, in combination with information on hatchery practices and hydrological connectivity, in order to delineate robust conservation and management units and to assess whether recent policy measures, such as the Yangtze fishing ban and improved hatchery practices, effectively stabilise or restore the remaining wild genetic resources of black carp.

## Figures and Tables

**Table 1:** Information on black carp samples used in this study, including site codes, localities, river basins and sample sizes.

River Basin	Code	Locality	Latitude (N)	Longitude (E)	Sample Size
Yangtze River, China	SS	Jingzhou, Hubei Province, China	29.562227°	112.396796°	12
	DTH	Yueyang, Hunan Province, China	29.374044°	113.095586°	17
	PYH	Duchang, Jiangxi Province, China	29.635389°	116.145810°	18
	WH	Hefei, Anhui Province, China	31.347710°	118.392009°	14
Pearl (Xijiang) River, China	GJ	Guilin, Guangxi Province, China	25.279783°	110.304321°	11
	FS	Foshan, Guangdong Province, China	23.178065°	112.903982°	21
Red River, Vietnam	YN	Hung Yen Province, Vietnam	20.646400°	106.051100°	7

**Table 2:** Genetic diversity, neutrality tests and mismatch-distribution statistics for black carp.

River Basin	Code	Number of Polymorphic Sites	Number of Haplotypes	Haplotype Diversity	Nucleotide Diversity (π)	Tajima’s D	Fu’s Fs	τ	Rg	SSD
Yangtze River	SS	5	5	0.576 ± 0.163	0.00106 ± 0.00036	−0.988	−1.418	2.118	0.129	0.012
	DTH	8	8	0.882 ± 0.047	0.00196 ± 0.00021	−0.196	−2.268	2.545	0.188 *	0.048 **
	PYH	6	4	0.634 ± 0.093	0.00172 ± 0.00025	0.408	1.558	3.447	0.324	0.084
	WH	6	7	0.912 ± 0.042	0.00198 ± 0.00023	0.696	−1.712	2.305	0.042	0.002
Yangtze River 4 groups		11	13	0.862 ± 0.022	0.00192 ± 0.00010	−0.189	−3.568	2.588	0.080 *	0.019 *
Pearl River	GJ	6	3	0.345 ± 0.172	0.00134 ± 0.00066	−1.007	1.491	5.026	0.477	0.022
	FS	9	6	0.724 ± 0.078	0.00251 ± 0.00026	0.495	0.867	4.515	0.204	0.041
Pearl River 2 groups		10	7	0.619 ± 0.086	0.00219 ± 0.00029	0.016	0.369	4.605	0.246	0.033
Red River-YN		4	3	0.524 ± 0.209	0.00100 ± 0.00052	−1.434	0.263	2.662	0.209	0.039
Total		16	17	0.826 ± 0.026	0.00208 ± 0.00010	−0.655	−5.218 *	2.971	0.076	0.021

Note: Significance levels are indicated by * *p* < 0.05, ** *p* < 0.01. For Tajima’s D and Fu’s Fs, significant values (with asterisks) indicate departures from neutral mutation–drift equilibrium. For the mismatch-distribution statistics, SSD (sum of squared deviations between observed and expected mismatch distributions) and Rg (Harpending’s raggedness index), the null hypothesis is a sudden demographic expansion model; non-significant values (no asterisks; *p* > 0.05) are consistent with a good fit to this model, whereas significant values (with asterisks; *p* < 0.05) indicate a poor fit.

**Table 3:** Pairwise FST and Nm among seven black carp populations.

Analysis	SS	DTH	PYH	WH	GJ	FS	YN
SS		2.064	1.019	3.945	0.518	2.428	0.349
DTH	0.195 **		11.710	90.409	1.988	12.752	1.946
PYH	0.329 ***	0.041		2.957	1.528	4.827	1.838
WH	0.112	0.006	0.145 *		2.555	28.452	1.766
GJ	0.491 ***	0.201 **	0.247 **	0.164 *		7.598	-
FS	0.171 *	0.038	0.094 *	0.017	0.062		-
YN	0.589 ***	0.204 *	0.214 *	0.221 *	−0.043	−0.071	

Note: Values below the diagonal are pairwise FST estimates; values above the diagonal are the corresponding estimates of gene flow (Nm). Asterisks indicate the significance of FST based on permutation tests: * *p* < 0.05; ** *p* < 0.01; *** *p* < 0.001. Slightly negative FST values can arise from sampling variance when within-population variation exceeds among-population variation; in biological terms, they were interpreted as indicating no detectable differentiation. For these pairs (e.g., GJ–YN and FS–YN), Nm was not calculated and is therefore shown as “-” in the table.

**Table 4:** Molecular variance analysis results of black carp population structure. Analysis of molecular variance (AMOVA) for mitochondrial Cyt b sequences of black carp.

Grouping Basis	Items	Among Groups	Among Populations Within Groups	Within Populations	Total	F Statistics (*p*-Value)
7 geographical populations	d.f.	6	*/*	93	99	*/*
	Sum of squares	22.459	*/*	100.767	123.226	*F*_st_ = 0.149 ***
	Variance components	0.189	*/*	1.084	1.273	*/*
	Percentage of variation	14.86	*/*	85.14	*/*	*/*
Yangtze River Group, Pearl River Group, Red River Group	d.f.	2	4	93	99	*F*_ct_ = 0.058
	Sum of squares	9.605	12.854	100.767	123.226	*F*_sc_ = 0.116 ***
	Variance components	0.076	0.142	1.084	1.302	*F*_st_ = 0.168 ***
	Percentage of variation	5.82	10.94	83.24	*/*	*/*
Yangtze River Group, Pearl River-Red River Group	d.f.	1	5	93	99	*F*_ct_ = 0.075 *
	Sum of squares	7.533	14.027	95.590	117.150	*F*_sc_ = 0.112 ***
	Variance components	0.094	0.129	1.028	1.251	*F*_st_ = 0.179 ***
	Percentage of variation	7.53	10.34	82.14	*/*	*/*
Group 1 (GJ, YN), Group 2 (SS, DTH, PYH, WH, FS)	d.f.	1	5	93	99	*F*_ct_ = 0.088 *
	Sum of squares	9.747	15.598	119.478	144.822	*F*_sc_ = 0.222 **
	Variance components	0.241	0.125	1.285	1.650	*F*_st_ = 0.146 ***
	Percentage of variation	14.60	7.56	77.84	*/*	*/*

Note: Results are shown for four hierarchical grouping schemes: (i) all seven populations analysed without higher-level grouping, (ii) populations grouped by drainage basin (Yangtze, Pearl and Red rivers), (iii) two drainage groups contrasting the Yangtze basin with the combined Pearl–Red river basins, and (iv) two groups defined by the best SAMOVA solution (Group 1: GJ and YN; Group 2: SS, DTH, PYH, WH and FS). Significance levels: * *p* < 0.05; ** *p* < 0.01; *** *p* < 0.001.

## Data Availability

The mitochondrial haplotype sequences of *Mylopharyngodon piceus* generated in this study have been deposited in the NCBI GenBank database under accession numbers PX894630–PX894646.

## Abbreviations

The following abbreviations are used in this manuscript:

Cyt b	cytochrome b
SS	Shishou
DTH	Dongtinghu
PYH	Poyanghu
WH	Wuhu
GJ	Guijiang
FS	Foshan
YN	Hung Yen (Red River, Vietnam)
Hd	haplotype diversity
π	nucleotide diversity
AMOVA	analysis of molecular variance
FST	fixation index (genetic differentiation coefficient)
Nm	gene flow among populations
SSD	sum of squared deviations
R_g_	Harpending’s raggedness index
